# Pseudogene AK4P1 promotes pancreatic ductal adenocarcinoma progression through relieving miR-375-mediated YAP1 degradation

**DOI:** 10.18632/aging.203921

**Published:** 2022-02-27

**Authors:** Lang Jia, Yun Zhang, Feng Pu, Chong Yang, Shula Yang, Jinze Yu, Zihan Xu, Hongji Yang, Yu Zhou, Shikai Zhu

**Affiliations:** 1 Organ Transplant Center, Sichuan Provincial People’s Hospital, University of Electronic Science and Technology of China, Chengdu 610072, China; 2 Clinical Immunology Translational Medicine Key Laboratory of Sichuan Province, Sichuan Provincial People’s Hospital, University of Electronic Science and Technology of China, Chengdu 611731, China; 3 Human Disease Gene Study Key Laboratory of Sichuan Province, Sichuan Provincial People’s Hospital, University of Electronic Science and Technology of China, Chengdu 610072, China; 4 Chinese Academy of Sciences Sichuan Translational Medicine Research Hospital, Chengdu 610072, China; 5 School of Clinical Medicine, Southwest Medical University, Luzhou 646000, China

**Keywords:** pancreatic ductal adenocarcinoma, pseudogene, AK4P1, miR-375, ceRNA

## Abstract

Pseudogenes have been reported to play oncogenic or tumor-suppressive roles in cancer progression. However, the molecular mechanism of most pseudogenes in pancreatic ductal adenocarcinoma (PDAC) remains unknown. Herein, we characterized a novel pseudogene-miRNA-mRNA network associated with PDAC progression using bioinformatics analysis. After screening by dreamBase and GEPIA, 12 up-regulated and 7 down-regulated differentially expressed pseudogenes (DEPs) were identified. According to survival analysis, only elevated AK4P1 indicated a poor prognosis for PDAC patients. Moreover, we found that AK4 acts as a cognate gene of AK4P1 and also predicts worse survival for PDAC patients. Furthermore, 32 miRNAs were predicted to bind to AK4P1 by starBase, among which miR-375 was identified as the most potential binding miRNA of AK4P1. A total of 477 potential target genes of miR-375 were obtained by miRNet, in which 49 hub genes with node degree ≥ 20 were identified by STRING. Subsequent analysis for hub genes demonstrated that YAP1 may be a functional downstream target of AK4P1. To confirmed the above findings, microarray, and qRT-PCR assay revealed that YAP1 was dramatically upregulated in both PDAC cells and tissues. Functional experiments showed that knockdown of YAP1 significantly suppressed PDAC cells growth, increased apoptosis, and decreased the ability of invasion. In conclusion, amplification of AK4P1 may fuel the onset and development of PDAC by targeting YAP1 through competitively binding to miR-375, and serve as a promising biomarker and therapeutic target for PDAC.

## INTRODUCTION

Pancreatic ductal adenocarcinoma (PDAC) has been the seventh major cause of cancer-related deaths worldwide with an estimated 466 003 deaths occurring in 2020 [1]. Despite the development of novel screening and therapeutic strategies in recent decades, the prognosis of PDAC patients is not optimistic, and the 5-year survival rate remains less than 9% [2–4]. The high incidence-to-mortality ratio of PDAC is mainly due to early tumor spread, lack of effective therapies, and prone to recurrence and metastasis after surgery [3]. Thus, comprehensive understandings of PADC pathogenesis are urgently needed, which are helpful to provide novel promising diagnostic biomarkers and effective therapeutic targets, and improve the prognosis of PADC patients.

Pseudogenes, belong to a class of long noncoding RNAs (lncRNAs), were traditionally regarded as non-functional “junk genes” owing to lack of protein-coding ability [5–7]. Recent studies have demonstrated that pseudogenes, as competing endogenous RNAs (ceRNAs), can exert a variety of biological functions by competitively binding to shared miRNAs of their downstream target genes [8–11]. Although genomic DNA sequences of pseudogenes were similar to their parental genes, the expression levels of parental genes also could affect pseudogenes’ expression [12]. To date, over 13,000 annotated pseudogenes have been identified [13, 14]. Increasing evidence have reported that the majority of pseudogenes play oncogenic or tumor-suppressive roles in various cancers, and also maybe act as promising diagnostic biomarkers and effective therapeutic targets [15, 16]. Recent studies have highlighted the importance of pseudogenes in gene regulation, which may ultimately affect many aspects of tumorigenesis and the development of cancers, including PDAC [17–20]. For instance, pseudogene DUXAP8 promotes PDAC cells growth by epigenetically silencing CDKN1A and KLF2 [21]. Knockdown of DUXAP10 inhibits PDAC cells proliferation and invasion, and promotes cells apoptosis through interacting with EZH2 and LSD1 [22]. Likewise, depletion of pseudogene ZFP91P significantly decreased PDAC cells proliferation and migration capacities by altering beta-catenin and vimentin expression [23]. SUMO1P3 could promote PDAC cells proliferation, migration, and invasion via the EMT signaling pathway [24]. PTTG3P promotes the progression of PDAC by miR-132/212-3p/FoxM1 signaling pathway [25]. Recent studies identified that pseudogenes AC093616.1, AC009951.1, TMEM183B and PABPC1P4 played critical roles in PDAC metastasis through regulating miR-30d-5p/GJA1 axis [26]. However, the pseudogene-miRNA-mRNA networks in PDAC have not yet been fully elucidated.

In the present study, we constructed a novel pseudogene-miRNA-mRNA network related to the progression of PADC through a series of bioinformatics analyses. Firstly, we acquired differentially expressed pseudogenes (DEPs) in PDAC through dreamBase databases. Furthermore, we screened those survival-related DEPs by the GEPIA databases. Based on the correlation and survival analysis, miR-375 potential binding to pseudogene AK4P1 was identified by using starBase databases. Next, the potential upstream dysregulated mechanisms (pseudogene-miRNA-mRNA network) were further explored using miRNet and STRING. Finally, the above analytic results were validated by microarray, qRT-PCR, CCK-8, flow cytometry, and transwell assays. In this study, we first introduce the AK4P1-miR-375-YAP1 ceRNA network using the bioinformatic tools, which may enable us to have a more comprehensive understanding of PDAC pathogenesis, and also provide a novel diagnostic biomarker and therapeutic target for PDAC.

## RESULTS

### Identification of AK4P1 as a potential prognostic regulator in PDAC

**Figure 1:**
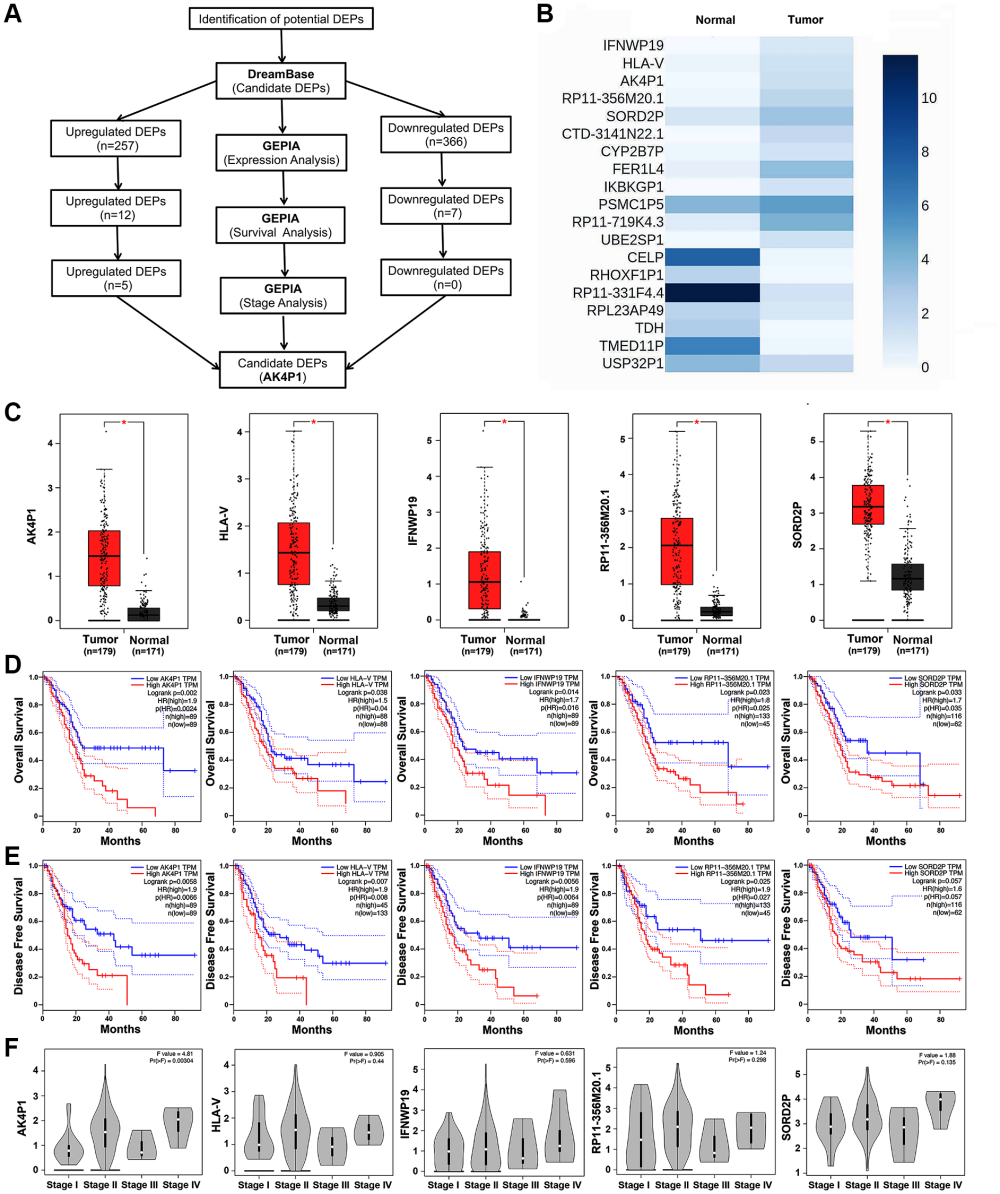
**Identification of pseudogene AK4P1 as a potential regulator in PDAC.** (**A**) The flow-process diagram for screening pseudogene AK4P1. (**B**) Expression of 19 potential DEPs in PDAC and normal pancreas tissues from TCGA and GTEx data. (**C**) Expression of 5 potential DEPs in PDAC and normal pancreas tissues. (**D**) Prognostic role (OS) of 5 potential DEPs in PDAC. (**E**) Prognostic role (DFS) of 5 potential DEPs in PDAC. (**F**) Expression of 5 potential DEPs among major stages in PDAC. Abbreviation: HR: hazard ratio; Three horizontal lines in the box plot represent minimum, median and maximum, respectively; ^*^P < 0.05.

To evaluate the clinical roles of pseudogenes in PDAC, a series of bioinformatic tools were conducted to identify potential DEPs (Figure 1A). All DEPs were first obtained to explore the functional pseudogenes by dreamBase [27]. Based on the cut-off criteria (|log_2_FC| >1.0), 623 potential DEPs (including 257 upregulated and 366 downregulated DEPs) were finally identified in PDAC (Supplementary Table 1). Next, to confirm the above preliminary data, the expression levels of these DEPs were validated by GEPIA [28]. We found that 12 upregulated and 7 downregulated DEPs were consistent with the above data from dreamBase (Figure 1B and Supplementary Figure 1). Then, the prognostic roles of the above 19 DEPs in PDAC were studied by GEPIA (Table 1). Five upregulated DEPs (AK4P1, IFNWP19, HLA-V, RP11-356M20.1, and SORD2P) indicated poorer overall survival (OS) for PDAC patients, and except SORD2P, other four DEPs indicated poorer disease-free survival (DFS) (Figure 1C–1E). On the other hand, none of the 7 downregulated DEPs affects the prognosis of PDAC patients (Table 1 and Supplementary Figure 2). Moreover, expression analysis of the 5 upregulated DEPs among tumor node metastasis (TNM) stages suggested that only AK4P1 expression possessed statistical difference (Figure 1F). Taken together, pseudogene AK4P1 may serve a crucial role in PDAC tumorigenesis.

**Table 1:** Prognostic roles of 17 potential pseudogenes in pancreatic cancer determined by GEPIA.

**Pseudogenes**	**Expression (Tumor/Normal)**	**OS**		**DFS**		**TNM Stage**	
		**HR**	***P*-value**	**HR**	***P*-value**	**F-value**	***P*-value**
IFNWP19	upregulated	1.7	0.016	1.9	0.006	0.63	0.596
HLA-V	upregulated	1.5	0.038	1.9	0.007	0.91	0.44
AK4P1	upregulated	1.9	0.002	1.9	0.007	4.81	0.003
RP11-356M20.1	upregulated	1.9	0.027	1.8	0.025	1.24	0.298
SORD2P	upregulated	1.7	0.033	1.6	0.057	1.88	0.135
CTD-3141N22.1	upregulated	1	0.84	1.3	0.32	0.06	0.979
CYP2B7P	upregulated	0.9	0.6	0.59	0.23	1.48	0.221
FER1L4	upregulated	1.3	0.22	1.3	0.25	3.53	0.016
IKBKGP1	upregulated	1.2	0.4	1.3	0.2	1.19	0.313
PSMC1P5	upregulated	0.83	0.37	1.1	0.56	2.21	0.089
RP11-719K4.3	upregulated	1.3	0.16	0.99	0.95	4.62	0.004
UBE2SP1	upregulated	1.1	0.75	1.2	0.44	0.85	0.47
CELP	downregulated	1.1	0.57	0.85	0.47	1.57	0.199
RHOXF1P1	downregulated	0.94	0.75	1.2	0.45	2.17	0.094
RP11-331F4.4	downregulated	1.1	0.52	0.92	0.7	1.65	0.18
RPL23AP49	downregulated	0.71	0.1	0.85	0.46	0.33	0.804
TDH	downregulated	0.88	0.53	0.81	0.37	1.15	0.332
TMED11P	downregulated	1.1	0.75	0.83	0.39	1.43	0.235
USP32P1	downregulated	0.84	0.4	0.97	0.89	0.14	0.937

### AK4 acts as a cognate gene of AK4P1 to predict worse survival in PDAC

**Figure 2:**
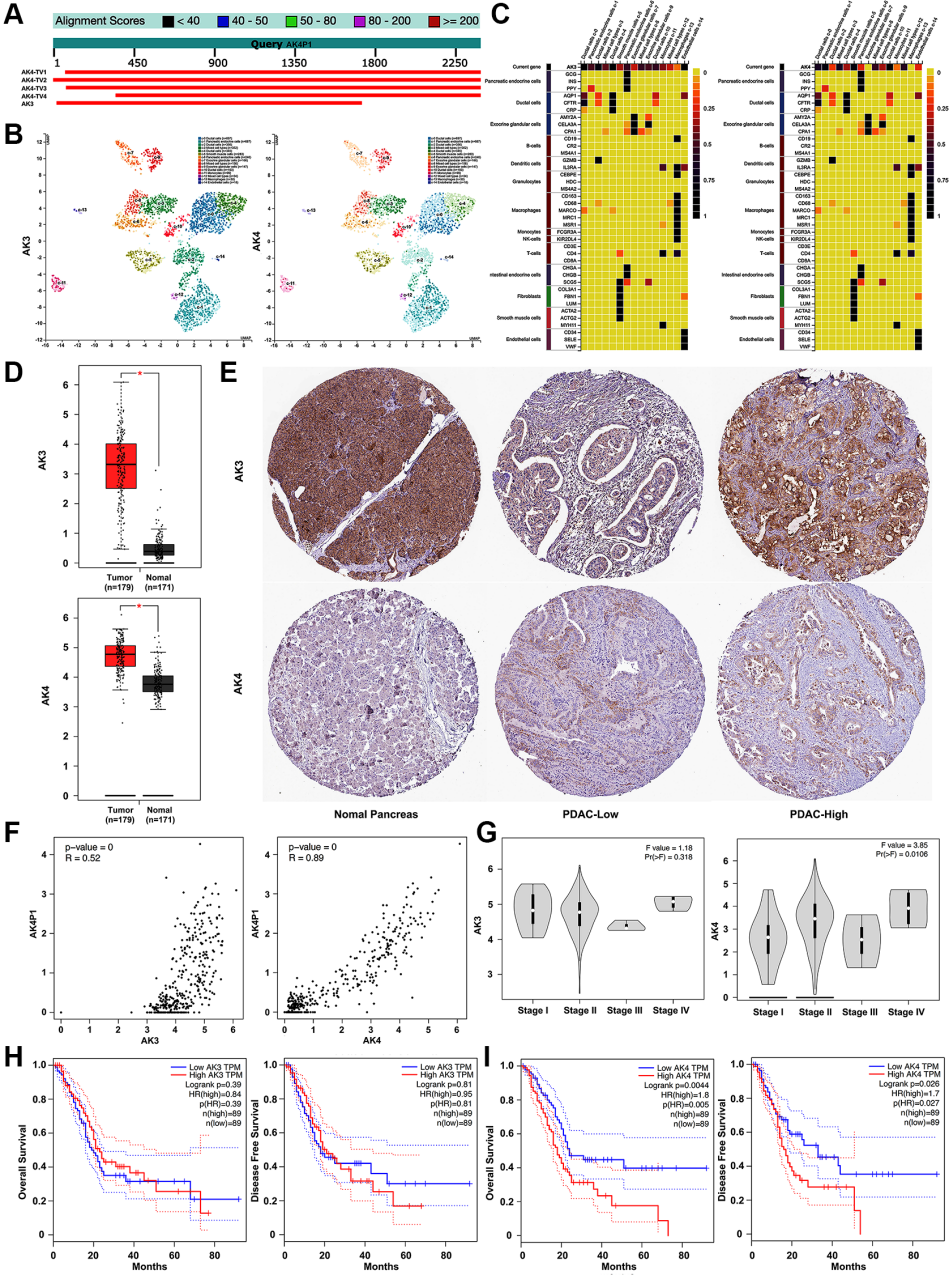
**Exploration of the relationship between AK4P1 and cognate genes in PDAC.** (**A**) Two cognate genes (AK3 and AK4) of pseudogene AK4P1. (**B**) The distribution of AK3/4 gene in different cell types of human normal pancreas was confirmed by scRNA-seq. (**C**) The expression level of the AK3/4 gene in different cell types of normal pancreas was determined by scRNA-seq. (**D**) The mRNA levels of AK3/4 expression in PDAC and normal pancreas tissues were determined by RNA-seq data from GEPIA. (**E**) The protein levels of AK3/4 expression in PDAC and normal pancreas tissues were determined by immunohistochemical staining data from HPA. (**F**) The expression correlation between AK4P1 and its cognate genes in PDAC was assessed using GEPIA. (**G**) Expression of AK3/4 genes among major stages in PDAC. (**H**) Prognostic role (OS) of AK3/4 genes in PDAC. (**I**) Prognostic role (DFS) of 5 potential DEPs in PDAC. Three horizontal lines in the box plot represent minimum, median and maximum, respectively; scale bar, 100 mm; ^*^P < 0.05.

Accumulating evidence documented that most pseudogenes could interact with their cognate genes [29]. Herein, we preliminarily explored the cognate genes of pseudogene AK4P1. Two potential cognate genes (AK3/4) of AK4P1 were found using BLAST (Figure 2A). Subsequently, the distribution and expression of AK3/4 genes in different cell types of human pancreas were confirmed by the single-cell RNA sequencing (scRNA-seq) data from the human protein atlas (HPA) database [30]. The scRNA-seq data showed that AK3 gene was dominantly expressed in ductal cells and endothelial cells, while AK4 gene was mainly expressed in ductal cells, smooth muscle cells, and pancreatic endocrine cells (Figure 2B, 2C). The mRNA levels of AK3/4 expression were analyzed by GEPIA. The results showed that the expression levels of AK3/4 were markedly upregulated in PDAC tissues compared with that in normal pancreas tissues (Figure 2D). On the other hand, the protein levels of AK3/4 expression were determined by immunohistochemical staining data from HPA. Only AK4 protein was markedly increased in PDAC tissues compared with that in normal pancreas tissues (Figure 2E). Next, the correlation between AK4P1 and its cognate gene was analyzed using GEPIA. Two potential cognate genes (AK3/4) were significantly positively associated with AK4P1 (R = 0.52 and R = 0.89, respectively) (Figure 2F). However, the expression of AK3/4 among major tumor stages demonstrated that only AK4 possessed a statistical difference (Figure 2G). Moreover, survival analysis found that only PDAC patients with higher levels of AK4 expression had poorer OS and DFS (Figure 2H and 2I). Taken together, AK4 acts as a cognate gene of AK4P1 to predict worse survival for PDAC patients. However, the regulatory effect of AK4P1 on AK4 in PDAC needs to be further experimentally validated.

### miR-375 is a potential binding miRNA of AK4P1 in PDAC

**Figure 3:**
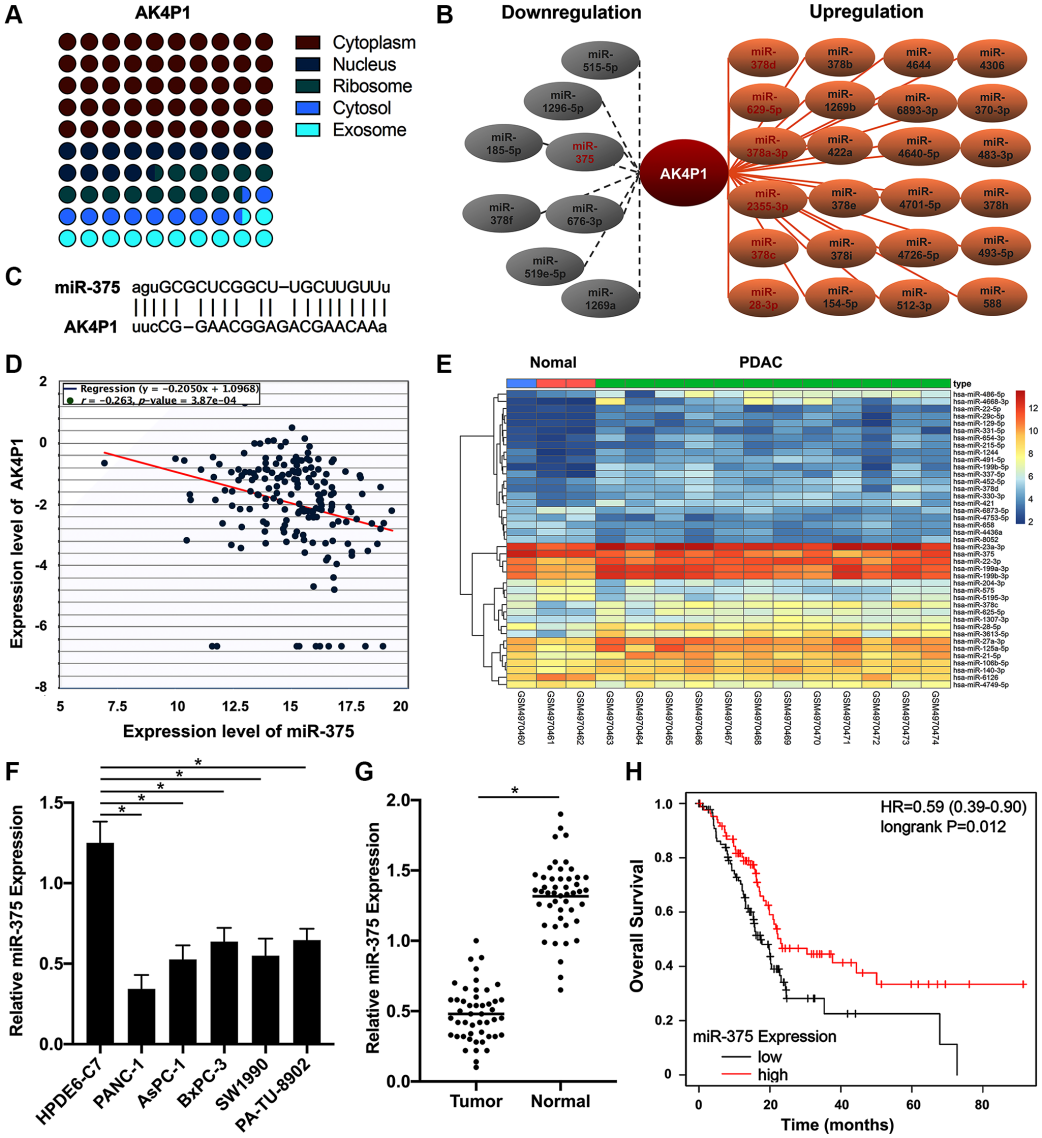
**miR-375 as a potential binding miRNA of AK4P1 in PDAC.** (**A**) The cellular location of AK4P1 is predicted by lncLocator. (**B**) Establishment of the potential AK4P1-miRNA regulatory network in PDAC, the red letter represents *P*-value less than 0.05. (**C**) Potential binding map between miR-375 and AK4P1. (**D**) The correlation of miR-375 and AK4P1 expression in PDAC. (**E**) Heatmap of miR-375 in PDAC tissues compared to normal pancreas tissues was assessed using microarrays data from GEO databases. (**F**) Expression of miR-375 in PDAC cell lines and normal pancreas cell lines. (**G**) Expression of miR-375 in 48 paired PDAC and their adjacent non-cancerous tissues. (**H**) The prognostic value (OS) of miR-375 expression in PDAC was assessed by TCGA databases.

CeRNA hypothesis has been widely documented to act as an important regulatory mechanism for serval diffident types of noncoding RNAs, such as lncRNAs, circRNAs, and pseudogenes [31, 32]. To ascertain whether pseudogene AK4P1 played its roles in PDAC by ceRNA hypothesis, we first investigated the subcellular location of AK4P1 by lncLocator [33]. The results showed that AK4P1 was mainly located in the cytoplasm (49.82%) and cytosol (10.22%) (Figure 3A). Subsequently, 32 potential miRNAs binding to AK4P1 were predicted by starBase (Supplementary Table 2). Among these 32 miRNAs, 8 downregulated miRNAs and 24 upregulated miRNAs were found in PDAC (Figure 3B). According to the ceRNA hypothesis, AK4P1 was negatively correlated with potential miRNA. Among these downregulated miRNAs, only the miR-375-AK4P1 pair was demonstrated a significantly negative relationship in PDAC (R = −0.263, *P* = 0.000387) (Figure 3C, 3D). Moreover, microarray analysis from GEO databases (GSE163031) displayed that the expression levels of miR-375 were markedly increased in PDAC tissues compared with that in normal pancreas tissues (Figure 3E). To confirmed those above results, we also examined by the qRT-PCR method that the levels of miR-375 expression in five PDAC cell lines (PANC-1, AsPC-1, BxPC-3, SW1990, and PA-TU-8902) were significantly downregulated compared with that in one normal pancreatic ductal epithelial cell line (HPDE6-C7) (Figure 3F). And similar results were confirmed by qRT-PCR in 48 paired PDAC tissues and non-cancerous tissues (Figure 3G). Furthermore, survival analysis using TCGA databases confirmed that only PDAC patients with a higher level of miR-375 expression had a better prognosis (HR = 0.59, 95% CI: 0.39–0.90, *P* = 0.012, Figure 3H), supporting that miR-375 is a potential binding miRNA of AK4P1 in PDAC.

### Screening for potential downstream target genes of the AK4P1/miR-375 axis

**Figure 4:**
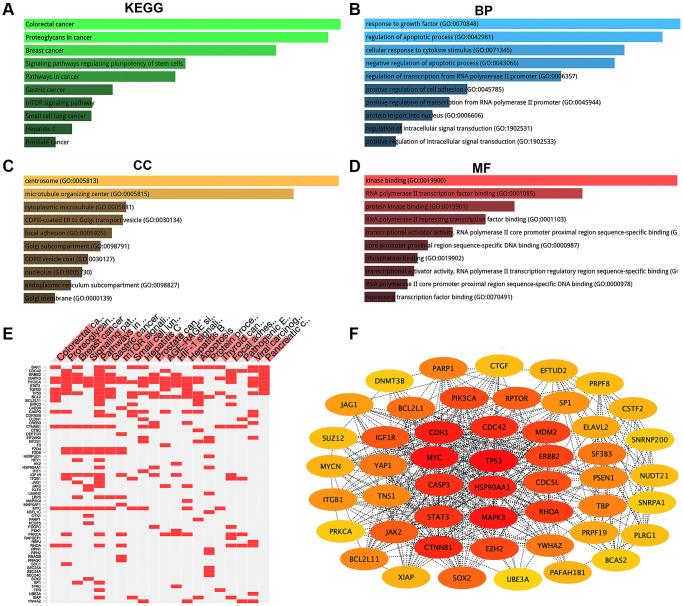
**KEGG pathway enrichment, GO functional annotation, and PPI network analysis for target genes of miR-375.** (**A**) The top 10 enriched KEGG pathway items. (**B**) The top 10 enriched biological process (BP) items. (**C**) The top 10 enriched cellular component (CC) items. (**D**) The top 10 enriched molecular function (MF) items. (**E**) The target genes of the top 20 enriched KEGG pathway. (**F**) The top 49 hub genes (node degree ≥ 20) in the PPT network of target genes.

To explore downstream targets of the AK4P1/miR-375 axis, we first predicted the potential target genes of miR-375 by miRNet [34]. 477 potential downstream target genes of miR-375 were obtained (Supplementary Table 3). Furthermore, the above target genes were mapped into the Enrichr database, and then KEGG pathway enrichment analysis and GO functional annotation were performed. The results showed that those target genes were markedly enriched in several cancers and cancer-associated pathways, including colorectal cancer, proteoglycans in cancer, breast cancer, pathways in cancer, and gastric cancer (Figure 4A and 4E). On the other hand, many significant enriched GO terms were found, including response to growth factor (GO:0070848), regulation of apoptotic process (GO:0042981), and cellular response to cytokine stimulus (GO:00471345) in the biological process (BP) category (Figure 4B); centrosome (GO:0005813), microtubule organizing center (GO:0005815), and cytoplasmic microtubule (GO:0005881) in the cellular component (CC) category (Figure 4C); kinase binding (GO:0019900), RNA polymerase II transcription factor binding (GO:0001085), and protein kinase binding in the molecular function (MF) category (GO:0019901) (Figure 4D). Furthermore, the PPI network of those potential target genes of miR-375 was established by STRING [35]. The results found that there were many gene-gene interactions among those target genes (Supplementary Table 4). Given that genes with higher nodes always have more functions, we calculated the node degree of each target gene by Cytoscape. Then the 49 eligible hub genes with node degree ≥ 20 were selected for subsequent analysis (Figure 4F).

### YAP1 as a target gene of AK4P1/miR-375 axis promotes PDAC progression

**Figure 5:**
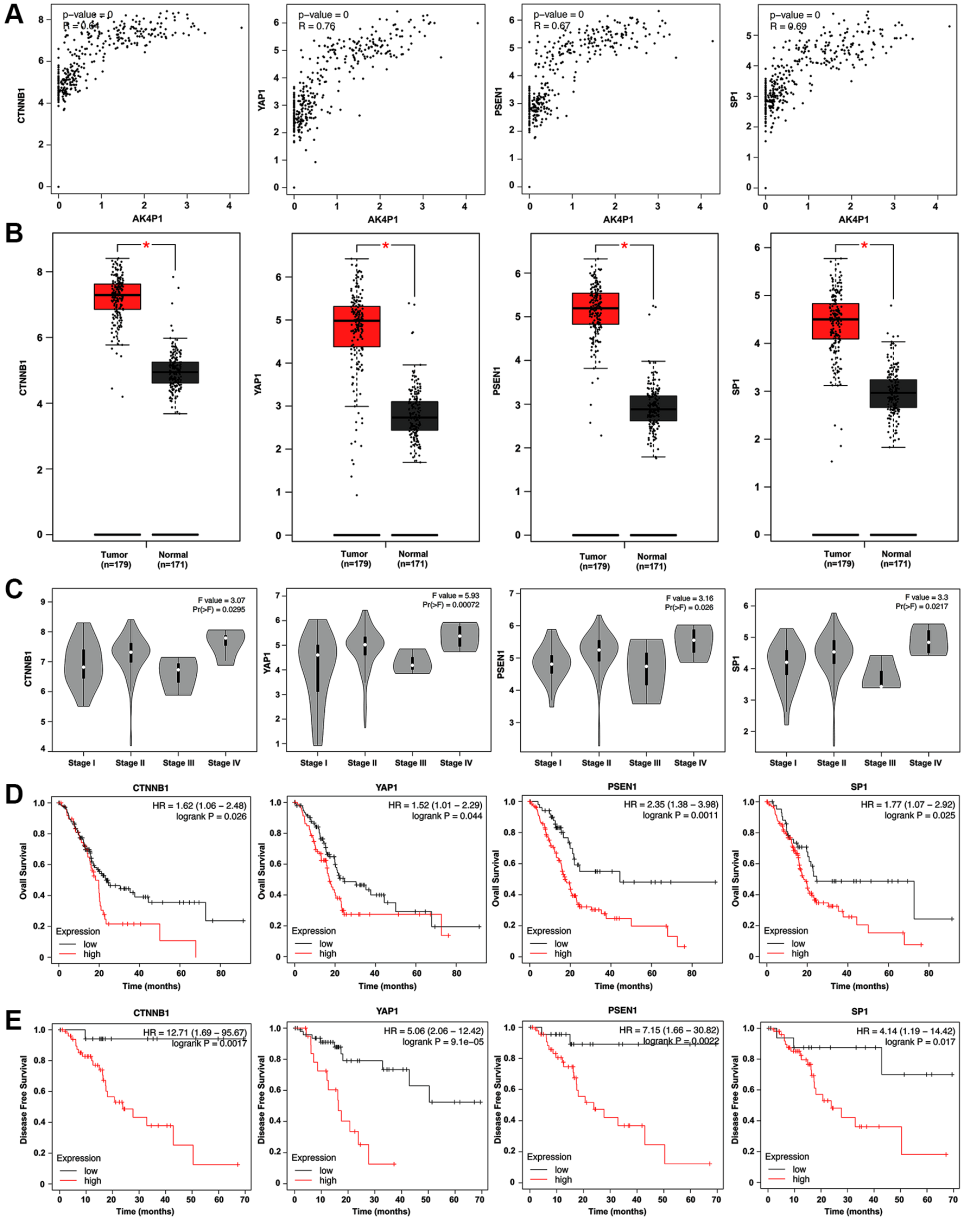
**Four hub genes are identified as functional target genes of AK4P1.** (**A**) The expression correlation between AK4P1 and four hub genes (CTNNB1, YAP1, PSEN1, and SP1) in PDAC was assessed using GEPIA. (**B**) Box-whisker plot represented expression of four hub genes (CTNNB1, YAP1, PSEN1, and SP1) in PDAC and normal pancreas tissues were determined by GEPIA. (**C**) Expression of four hub genes (CTNNB1, YAP1, PSEN1, and SP1) among major stages in PDAC were determined by GEPIA. (**D**) Prognostic role (OS) of four hub genes (CTNNB1, YAP1, PSEN1, and SP1) expression in PDAC. (**E**) Prognostic role (DFS) of four hub genes (CTNNB1, YAP1, PSEN1, and SP1) expression in PDAC. Three horizontal lines in the box plot represent minimum, median and maximum, respectively. ^*^P < 0.05.

**Figure 6:**
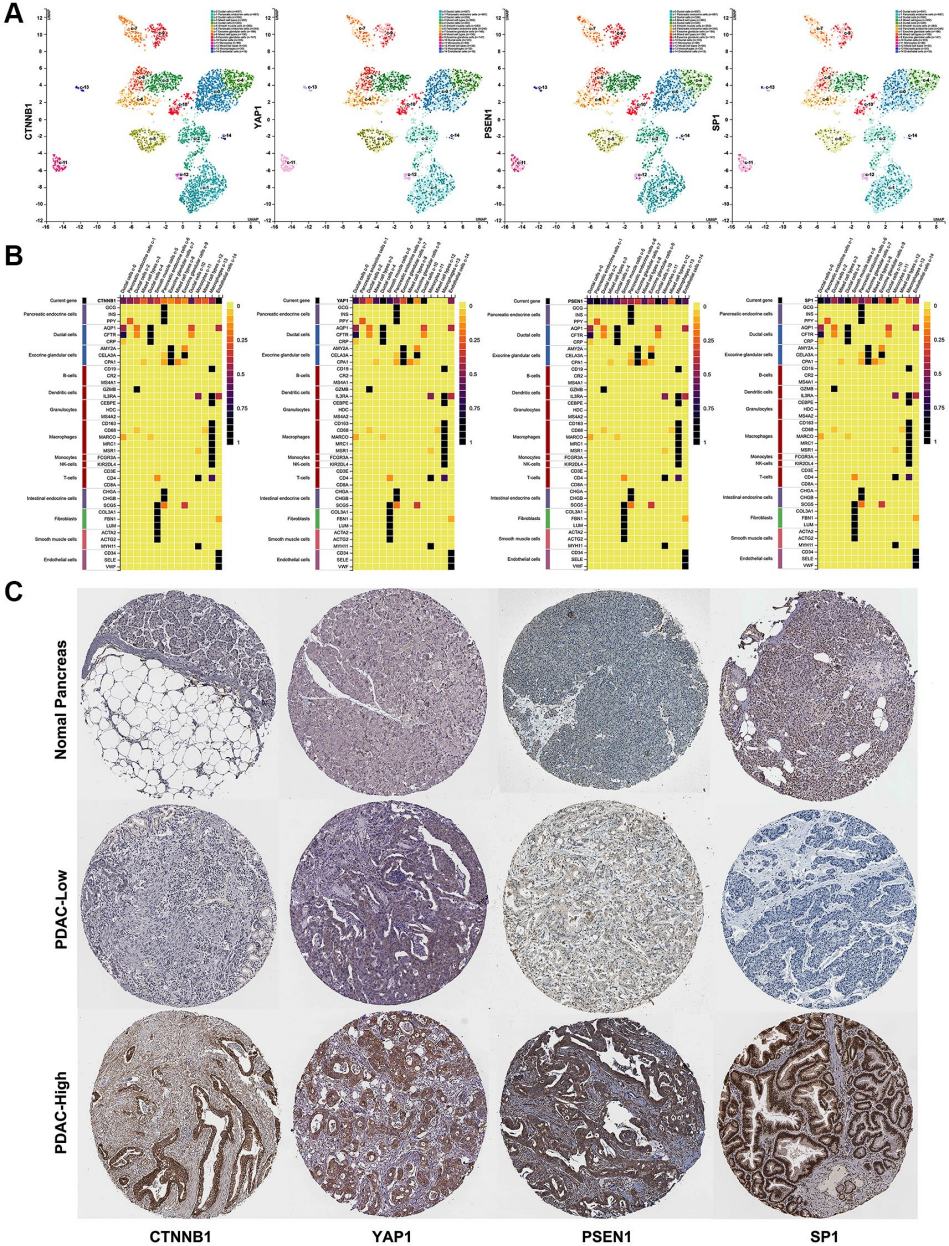
**The distribution and expression of four hub genes (CTNNB1, YAP1, PSEN1, and SP1) in PDAC.** (**A**) The distribution of four hub genes (CTNNB1, YAP1, PSEN1, and SP1) in different cell types of human pancreas was confirmed by scRNA-seq. (**B**) The expression level of four hub genes (CTNNB1, YAP1, PSEN1, and SP1) in different cell types of human pancreas were determined by scRNA-seq. (**C**) The protein levels of four hub genes (CTNNB1, YAP1, PSEN1, and SP1) expression in PDAC and normal pancreas tissues were determined by immunohistochemical staining data from HPA. scale bar, 100 mm.

Based on the ceRNA hypothesis, the expression level of AK4P1 should show a positive correlation with its potential functional target genes. Herein, we first studied the association between AK4P1 and the aforementioned hub genes. 13 of 49 hub genes were positively correlated with AK4P1 in PDAC (Table 2, Figure 5A). Then, the prognostic values of the 13 hub genes in PDAC were evaluated using GEPIA. The results demonstrated that four upregulated hub genes (CTNNB1, YAP1, PSEN1, and SP1) were markedly related to the TMN stage for PDAC patients (Figure 5B, 5C). Subsequently, the analytic results of the Kaplan-Meier plotter showed that overexpression of four hub genes indicated poorer OS and DFS for PDAC patients (Figure 5D, 5E). In addition, the distribution and expression of CTNNB1, YAP1, PSEN1, and SP1 in different cell types of human pancreas were also confirmed by scRNA-seq data from the HPA database [30]. The scRNA-seq data showed that all four hub genes were mainly expressed in ductal cells and endothelial cells, and PSEN1 and SP1 genes were also expressed in pancreatic endocrine cells (Figure 6A, 6B). Furthermore, the protein levels of CTNNB1, YAP1, PSEN1, and SP1 expression were determined by immunohistochemical staining data from HPA. All four hub genes protein in PDAC cancer tissues were markedly upregulated compared with that in normal pancreas tissues (Figure 6C).

**Table 2:** Prognostic values and correlation of hub genes with AK4P1 determined by Kaplan-Meier plotter and GEPIA.

**Hub genes**	**Expression**	**OS**			**RFS**			**AK4P1**		**TNM Stage**	
		**HR**	**95% CI**	***P*-value**	**HR**	**95% CI**	***P*-value**	**R**	***P*-value**	**F-value**	***P*-value**
CDH1	Up	1.86	1.14−3.03	0.012	3.73	1.38−10.09	0.0055	0.65	0	0.835	0.476
CTNNB1	Up	1.62	1.06−2.48	0.026	12.7	1.69−95.67	0.0017	0.64	0	3.07	0.0295
CASP3	Up	1.83	1.11−3.03	0.017	3.02	1.00−9.14	0.042	0.6	0	0.97	0.408
RHOA	Up	1.58	1.05−2.39	0.029	5.48	1.80−16.61	0.00093	0.65	0	2.02	0.112
PIK3CA	Up	1.94	1.23−3.05	0.0036	5.02	1.79−14.02	0.0008	0.73	0	1.96	0.122
YAP1	Up	1.52	1.01−2.29	0.044	6.06	2.06−12.42	0.000091	0.76	0	5.93	0.00072
PSEN1	Up	2.35	1.38−3.98	0.0011	7.15	1.66−30.82	0.0022	0.67	0	3.16	0.026
SP1	Up	1.77	1.07−2.92	0.025	4.14	1.19−14.42	0.017	0.69	0	3.3	0.0217
JAG1	Up	2.59	1.7−3.95	0.000004	1.67	2.78−33.59	0.000021	0.63	0	2.31	0.0779
CSTF2	Up	2.05	1.19−3.54	0.0081	4.01	1.16−13.93	0.019	0.66	0	1.56	0.201
EFTUD2	Up	1.98	1.16−3.41	0.011	4.03	1.19−13.6	0.015	0.52	0	1.4	0.244
BCAS2	Up	1.65	1.09−2.48	0.016	2.63	1.12−6.22	0.022	0.63	0	0.991	0.398
SNRNP200	Up	1.81	1.07−3.07	0.025	3.55	1.03−12.19	0.033	0.53	0	1.57	0.199

**Figure 7:**
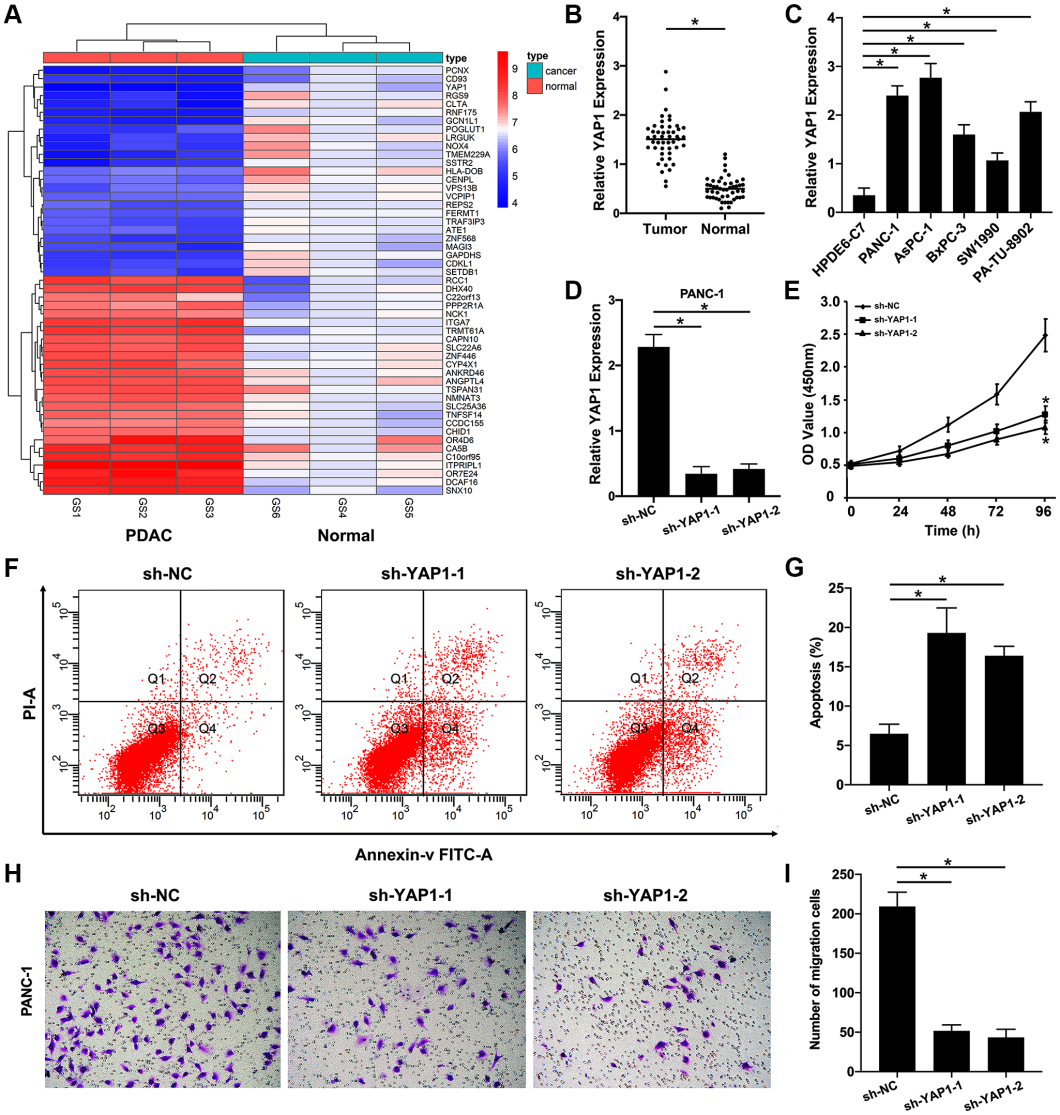
**YAP1 as a target gene of the AK4P1/miR-375 axis promotes PDAC progression.** (**A**) Heatmap of DEGs in PDAC tissues compared to normal pancreas tissues were confirmed by microarray. (**B**) Expression of YAP1 in PDAC and normal pancreas cell lines. (**C**) Expression of YAP1 in 48 paired PDAC and their adjacent non-cancerous tissues. (**D**) Expression of YAP1 in PANC-1 cells with stably transfected sh-YAP1-1 and sh-YAP1-2 were detected using qRT-PCR, and the densitometric values of YAP1 expression were analyzed as compared with GAPDH. (**E**) The effect of knocking down YAP1 on the proliferation of PANC-1 cells, according to the CCK-8 assay. (**F**) The effect of knocking down YAP1 on the apoptosis rate of PANC-1 cells was determined by flow cytometry. (**G**) The densitometric analysis of the apoptosis rate of PANC-1 cells after knocking down YAP1 was performed. (**H**) The effect of knocking down YAP1 on the ability of PANC-1 cells invasion was determined by Transwell assay. (**I**) The densitometric analysis of the number of the invasion cells after knocking down YAP1 was performed. Data are presented as the means ± SD. Results were one representative of three independent experiments; bars, SD; ^*^P < 0.05.

**Figure 8:**
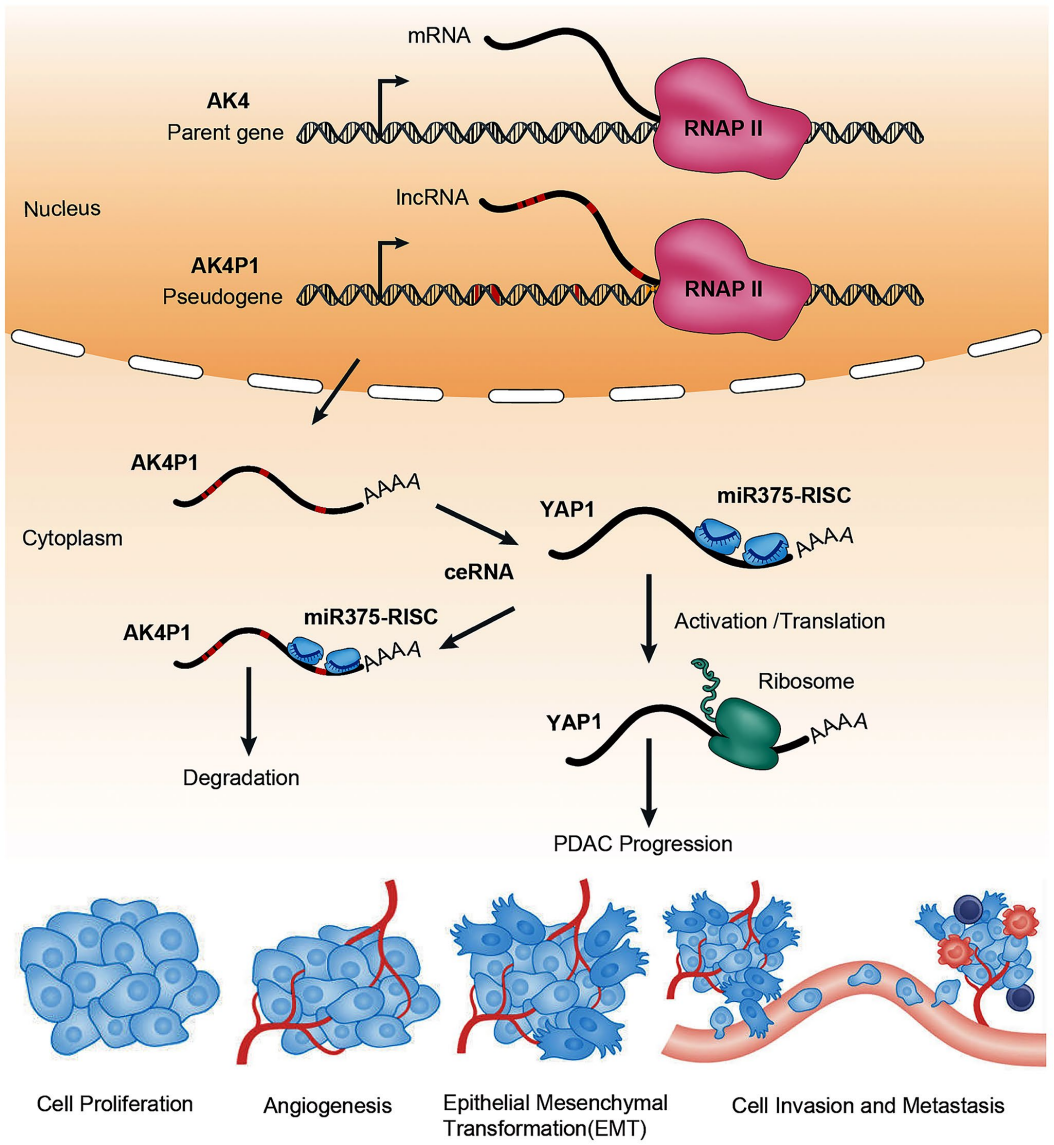
**The ceRNA mechanism of pseudogene AK4P1 promotes PDAC progression.** Genomic DNA sequence of pseudogene AK4P1 was similar to parent gene AK4. Where oncogene YAP1 is targeted by the miR375-guided RNA-induced silencing complex (miR375-RISC), leading to accelerate YAP1 degradation. And when the pseudogene-derived lncRNA is transcribed and exported to the cytoplasm, it competes for miRNA targeting and binding of RISC complexes, leading to relieve miR-375-mediated YAP1 degradation and increase YAP1 expression, which can promote PDAC progression. Abbreviations: AGO: Argonaute; RNAP II: RNA polymerase II.

To confirmed these bioinformatic analytic findings, our microarray analysis displayed that compared with normal pancreas tissues, the levels of YAP1 expression were markedly increased in PDAC tissues (Figure 7A). We also examined that the mRNA level of YAP1 expression in five PDAC cell lines (PANC-1, AsPC-1, BxPC-3, SW1990, and PA-TU-8902) were significantly downregulated compared with that in one normal pancreatic ductal epithelial cell line (HPDE6-C7) (Figure 7B). And the similar results were validated in 48 paired PDAC tissues and non-cancerous tissues (Figure 7C). Furthermore, loss-of-function studies revealed that knockdown of YAP1 could significantly suppress PDAC cells growth (Figure 7D, 7E), increased the rate of PDAC cells apoptosis (Figure 7F, 7G), and decreased the ability of PDAC cells invasion (Figure 7H, 7I). Taken together, these findings suggested that YAP1 may be the most potential functional target of pseudogene AK4P1 in PDAC (Figure 8).

## DISCUSSION

Pseudogenes are a special class of lncRNAs that have been often reported to play oncogenic or tumor-suppressive roles in various cancers [9, 18, 36–38]. Pseudogenes can regulate the downstream target genes expression through competitively binding to shared miRNAs [20, 26, 39]. However, most pseudogene-miRNA-mRNA ceRNA network in PDAC remains largely unknown. In this present study, we first reported a novel upregulated pseudogene AK4P1 in PDAC. Subsequent analysis demonstrated that a higher level of AK4P1 expression indicated poorer OS and DFS, and was also strongly associated with a worse tumor stage for PDAC patients. Moreover, we found that cognate gene AK4 was mainly expressed in ductal cells, and overexpression of AK4 also predict worse survival for PDAC patients. A similar role of AK4 in PDAC progression was found by Hao et al. [40]. Taken together, our results suggested that pseudogene AK4P1 may be a novel promising diagnostic biomarker and effective therapeutic target for PDAC.

Next, we explored the possible action mechanism of AK4P1 in PDAC. According to the ceRNA hypothesis, pseudogenes need to be localized in the cytoplasm so that they can be competitively bound to miRNA and thus disinhibit downstream targets [20, 41]. Firstly, we predicted by lncLocator that AK4P1 was mainly located in the cytoplasm [33]. Furthermore, we predicted potential miRNAs that theoretically bind to AK4P1 by starBase. 8 downregulated and 24 upregulated miRNAs that might bind to AK4P1 were screened in PDAC. The further study identified that miR-375 was a potential binding miRNA of AK4P1. In addition, survival analysis revealed that only PDAC patients with a higher level of miR-375 expression had a worse prognosis, supporting that miR-375 was a binding miRNA of AK4P1 in PDAC. In recent years, miR-375 has been reported to act as a tumor-suppressor miRNA in different types of cancers, including PDAC [42]. Upregulation of miR-375 could inhibit PDAC cells proliferation, migration, and chemosensitivity through binding to HOXB3 [43]. miR-375 also can target PDK1 and suppresses PDAC cells growth through the Akt pathway [44]. To sum up, our findings suggested that miR-375 was a potential binding miRNA of AK4P1 in PDAC.

To explore downstream targets of the AK4P1/miR-375 axis, we obtained 477 potential downstream target genes of miR-375 by miRNet. Furthermore, to explore the roles of AK4P1, we mapped the above target genes into the Enrichr database. The analytic results showed that those target genes were significantly enriched in several cancers and cancer-associated pathways including response to growth factors, and regulation of the apoptotic process. Given that genes with higher nodes always have more functions, the 49 eligible hub genes with node degree ≥ 20 were selected by STRING [35]. Based on the ceRNA hypothesis, the expression levels of AK4P1 should show a positive correlation with its potential functional target genes. After screening by the correlation and survival analysis, only four upregulated hub genes (CTNNB1, YAP1, PSEN1, and SP1) were included. Moreover, all four hub genes were mainly expressed in ductal cells and endothelial cells, and their protein levels in PDAC tissues were markedly increased compared with that in normal pancreas tissues. To confirm the aforementioned findings, we examined by microarray and qRT-PCR that only YAP1 was markedly upregulated in PDAC cells and tissues. YES-associated protein 1 (YAP1), a critical downstream effector of the Hippo-YAP pathway, has been found to promote PDAC development and progression, even in the absence of oncogenic gene *Kras* [45]. We thus performed loss-of-function studies, and the results revealed that knockdown of YAP1 could significantly suppress PDAC cells growth, increased the rate of PDAC cells apoptosis, and decreased the ability of PDAC cells invasion. Taken together, these findings suggested that YAP1 may be the most potential functional target of pseudogene AK4P1 in PDAC (Figure 8).

## CONCLUSIONS

In this study, we first introduce the AK4P1/miR-375/YAP1 ceRNA network in PDAC using bioinformatic tools. And validation experiments confirmed that amplification of pseudogenes AK4P1 played critical roles in facilitating PDAC progression through relieving miR-375-mediated YAP1 degradation. Moreover, those findings also suggested that AK4P1/miR-375/YAP1 ceRNA network possessed significant prognostic values for PDAC patients, and AK4P1 may serve as a novel diagnostic biomarker and effective therapeutic target for PDAC.

## MATERIALS AND METHODS

### Identification of DEPs related to prognosis in PDAC

The RNA-sequencing data of pseudogenes in PDAC were directly downloaded from dreamBase, which is an integrated online platform to analyze the regulatory functions of pseudogenes from high-throughput RNA-sequencing data [27]. The cut-off criterion for selecting DEPs was set at |log_2_FC| >1.0, FC = Tumor/Normal. Subsequently, the expressions levels of the above candidate DEPs were validated by gene expression profiling interactive analysis (GEPIA), which is an interactive online tool to analyze expression analysis, survival analysis, and correlation analysis [28]. *P*-value < 0.05 and |log_2_FC| >1.0 were set to identify potential prognostic DEPs. The expressions levels of DEPs among the TNM stage and the prognostic value of DEPs were analyzed by GEPIA.

### Prediction of subcellular localization of pseudogene AK4P1

The sequence of pseudogene AK4P1 was extracted from National Center for Biotechnology Information (NCBI). And then subcellular localization of pseudogene AK4P1 was explored by lncLocator [33], which could predict five main subcellular localizations of the pseudogene, namely the nucleus, cytoplasm, cytosol, ribosome, and exosome. Moreover, the figures derived from the above results were established by GraphPad Prism 8 (GraphPad Software Inc., CA, USA).

### Prediction of candidate miRNAs of pseudogene AK4P1

StarBase was utilized to predict the candidate miRNAs binding to pseudogene AK4P1, which is an online platform to study a variety of the RNA-RNA and RBP-RNA interactions [46]. The candidate miRNAs could be directly obtained, and then a pseudogene-miRNA network was visualized by Cytoscape 3.8 (Cytoscape Consortium , TX, USA). The correlation of miR-375 and AK4P1 expression in PDAC was analyzed by StarBase. For miR-375 analysis, microarray analysis from GEO databases (GSE163031) was included to study the expression level of miR-375 in PDAC tissues, and the Kaplan-Meier plotters using TCGA databases were used to determine the prognostic values of miR-375 in PDAC.

### Prediction of potential targets of miR-375

Potential downstream targets of miR-375 were predicted by miRNet, which is an online tool to provide statistical analysis and functional interpretation of published data on miRNAs [34]. KEGG pathway enrichment analysis for the targets of miR-375 was conducted by Enrichr [47]. GO functional annotation included three categories such as biological process (BP), cellular component (CC), and molecular function (MF) [47]. Furthermore, the protein-protein interaction (PPI) network for predicting target genes was built by STRING (http://string-db.org). Firstly, the candidate target genes were mapped into the STRING database. Next, the gene pairs with a combined score ≥ 0.4 were included. Finally, the hub genes among the PPI network were identified by cytoHubba plugin of Cytoscape 3.8 (Cytoscape Consortium, TX, USA). And the hub genes with node degree ≥ 20 were selected for subsequent analysis.

### The correlation between AK4P1 and hub genes

To determine potential targets of pseudogene AK4P1, the correlation between AK4P1 and hub gene in PDAC was analyzed by GEPIA. AK4P1-hub gene pairs with R > 0.1 and *P* < 0.05 were selected for further analysis. The prognostic values of hub genes were validated using GEPIA and Kaplan-Meier. Moreover, the distribution and location of hub genes in different cell types of human pancreas were assessed by scRNA-seq data from the HPA database [30], which is an open proteomic resource to study protein expression and patients’ prognosis.

### Patient tissue samples

A total of 48 freshly paired PDAC tissues and non-cancerous tissues were collected from Organ Transplant Center, Sichuan Provincial People’s Hospital (Chengdu, China) between 2019 and 2021. After surgical resection, all tissue samples were frozen in liquid nitrogen. All patients were diagnosed with PDAC following TNM stages. None received any chemoradiotherapy before surgery. This study was approved by the Ethics Committee of Sichuan Provincial People’s Hospital, and informed consent was obtained from all included patients.

### Cell culture

All seven cell lines were purchased from the Culture Collection of Chinese Academy of Sciences (Shanghai, China). The human pancreatic ductal epithelial cell lines (HPDE6-C7), and human renal epithelial cell line (HEK-293T) was maintained in DMEM medium with 10% fetal bovine serum (Gibco, USA), 1% penicillin, and streptomycin (Gibco, USA). PDAC cell lines (PANC-1, AsPC-1, SW1990, BxPC-3, and PA-TU-8902) were maintained in RPMI-1640 medium with 10% fetal bovine serum (Gibco, USA), 1% penicillin, and streptomycin (Gibco, USA). All seven cell lines were cultured in a humidified incubator containing 5% CO_2_ at 37°C.

### qRT-PCR

Total RNA was extracted using TRIzol reagent (Invitrogen, USA) according to the manufacturer’s instructions [48]. cDNA was synthesized using the Prime Script RT Reagent Kit (Takara, Japan). For miRNA, reverse transcriptions were performed using specific stem-loop primers. For mRNA, reverse transcriptions were done using random primers. cDNA amplification was detected using TB Green Premix Ex Taq II (Takara, Japan) with ABI Prism 7500 sequence detection system (Applied Biosystems, USA). The primers mentioned in this study were as follows: miR-375 forward: 5′-CCCTCTAGACCCCAAGGCTGATGCTGAGAAG-3′, reverse: 5′-AAAGGTCCGCCGCCCGGCCCCGGGTCTTC-3′; YAP1 forward:5′-CAGATGGAGAAGGAGAGGC-3′, reverse: 5′-ATTGATATTCCGCATTGCCTG-3′; and GAPDH forward: 5′-ACATCGCTCAGACACCATG-3′, reverse: 5′-TGTAGTTGAGGTCAATGAAGGG-3′. Relative quantification of miRNA and mRNA was compared to U6 and GAPDH, and was analyzed using the 2^−ΔΔCT^ method.

### Microarray

Three paired PDAC tissues and non-cancerous tissues were sent to microarray analysis using an Arraystar Human mRNA microarray. After removal of rRNA, mRNA was purified from total RNA (RNeasy Mini Kit, Qiagen). And mRNA was transcribed into fluorescent cRNA without 3’ bias along the entire length of the transcripts. Next, sample labeling was performed using Quick-Amp Labeling Kit (Agilent, CA, USA). Then, the specific activity and concentration of labeled cRNAs were determined by NanoDrop ND-1000. Finally, hybridization was done in an Agilent Hybridization Oven. The cut-off value = 2.0-fold and Benjamini-Hochberg corrected *P* < 0.05 were set to identify differentially expressed mRNAs.

### Cell transfection

Short hairpin RNAs (sh-RNAs) against YAP1 (sh-YAP1-1 and sh-YAP1-2), and negative control (sh-NC) were obtained from Gene Pharma (Shanghai, China). PANC-1 cells were put into 6-well plates until the density was 70–80% confluence. The above plasmids were separately transfected into PANC-1 cells using Lipofectamine 3000 (Invitrogen, USA). At 48 h after transfection, mature cells were gathered for further study.

### Cell proliferation assay

Cell Counting Kit-8 (CCK-8) was used to determine the ability of cell proliferation according to the manufacturer’s instructions. Briefly, PANC-1 cells were seeded on a 96-well plate in triplicates with 1000 cells/200 μl. At indicated time points (0 h, 24 h, 48 h, 72 h, and 96 h), 100 μl of fresh medium supplemented with 10 μl CCK-8 reagent (Beyotime, China) was added and incubated for an additional 3–4 h at 37°C. The absorbance at a wavelength of 450 nm was detected using a multiplate reader (Bio Tek, VT, USA).

### Apoptosis assay

Cell apoptosis was detected by the FITC Annexin V apoptosis Detection Kit (BD Biosciences, CA, USA). Briefly, PANC-1 cells (2 × 10^5^ cells/plate) in 6-well plates cells were incubated for 48 h. Then PANC-1 cells were collected by mild trypsinization. After being washed twice with cold PBS, PANC-1 cells were stained with FITC-Annexin V and propidium iodide (PI) on ice for 5 mins, and then subjected to a BD LSRFortessa analyzer (BD Biosciences, CA, USA).

### Transwell assay

The ability of cell invasion was detected using Transwell chambers (Corning, NY, USA) with Matrigel (BD Biosciences, CA, USA), according to the manufacturer’s instructions. Briefly, PANC-1 cells (5 × 10^4^ cells/plate) suspended in serum-free medium were plated in the upper chambers. And then medium supplemented with 10% FBS was placed in the lower chambers. After incubation for 24 h, the cells in the upper chambers were stained by crystal violet (Kaigen, China) for 15 mins, and then the invasion cells were photographed and counted in different five random fields.

### Statistical analysis

Data were presented as mean ± standard deviation (SD) of triplicate biological replicates or samples. The statistical analysis of bioinformatics data was directly performed by various online tools. GraphPad Prism 8 (GraphPad Software, CA, USA) was applied to analyze all experimental data in this study. The statistical significance for qualitative data and quantitative data were analyzed by Chi-square test and two-tailed Student’s respectively. All statistical analyses were performed using the SPSS software package ver20.0 (SPSS, Inc., IL, USA). *P* < 0.05 was considered statistically significant.

## Supplementary Materials

Supplementary Figures

Supplementary Table 1

Supplementary Table 2

Supplementary Table 3

Supplementary Table 4
