# Supplementary material for: Pseudogene AK4P1 promotes pancreatic ductal adenocarcinoma progression through relieving miR-375-mediated YAP1 degradation
Source: Aging (Albany NY). 2022 Feb 27;14(4):1983–2003. doi: 10.18632/aging.203921 (PMC8908928; doi:10.18632/aging.203921)
Supplement: Supplementary Figures [file aging-14-203921-s001.pdf]

SUPPLEMENTARY FIGURES

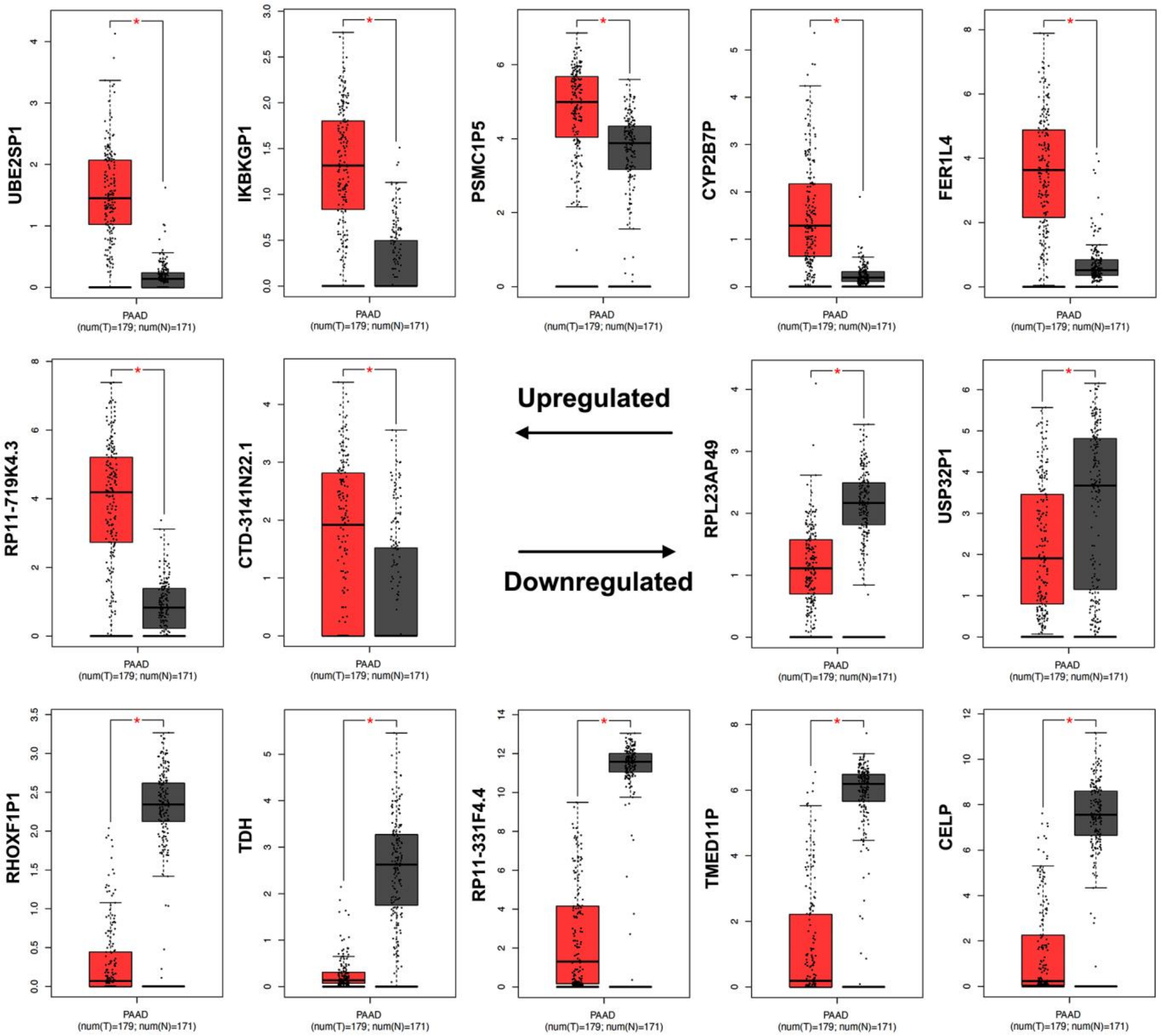

Supplementary Figure 1. Expression of 14 potential DEPs in PDAC. Expression of 14 potential DEPs in PDAC and normal pancreas tissues form TCGA and GTEx data. Three horizontal lines in the box plot represent minimum, median and maximum, respectively; $^{*}P < 0.05$.

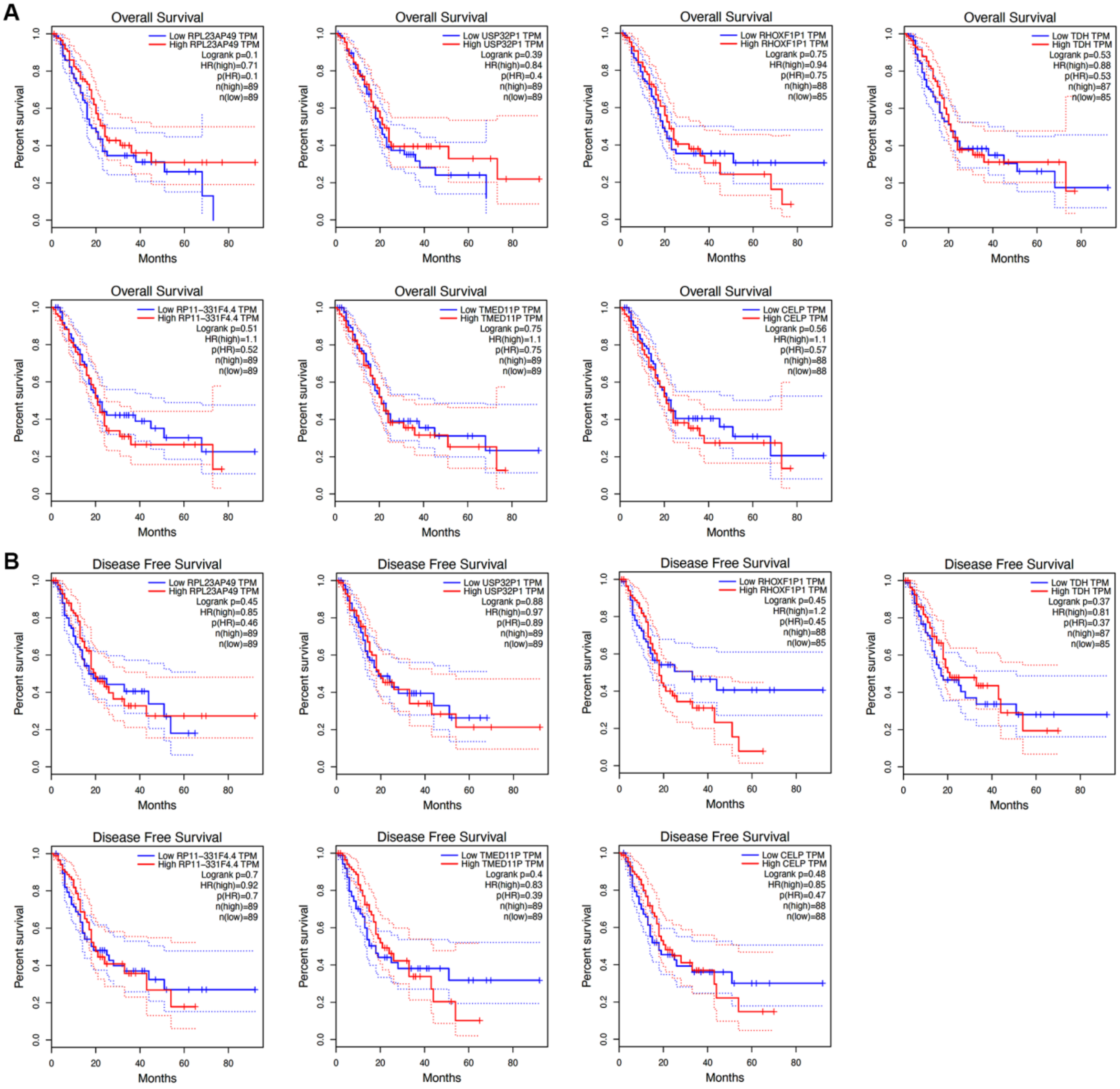

Supplementary Figure 2. Prognostic role of 7 potential upregulated DEPs in PDAC. (**A**) Prognostic role (OS) of 7 potential upregulated DEPs in PDAC. (**B**) Prognostic role (DFS) of 7 potential upregulated DEPs in PDAC.
